# Effects of astrocytic PKM2 gene deletion on neuronal death following traumatic brain injury

**DOI:** 10.1038/s41420-025-02829-7

**Published:** 2025-11-10

**Authors:** Beom Seok Kang, Min Kyu Park, Hyun Wook Yang, Dong Yeon Kim, Hyun Ho Jung, Aileen Song, Jaewoo Shin, Dae-Soon Son, Bo Young Choi, Sang Won Suh

**Affiliations:** 1 Department of Physiology, Hallym University, College of Medicine, Chuncheon, Republic of Korea; 2 Department of Pharmacology and Neuroscience, Creighton University School of Medicine, Omaha, NE USA; 3 Department of Biology, University of Washington, College of Arts and Sciences, Seattle, WA USA; 4 Medical Device Development Center, Daegu-Gyeongbuk Medical Innovation Foundation (K-MEDI Hub), Daegu, Republic of Korea; 5 Division of Data Science, Data Science Convergence Research Center, Hallym University, Chuncheon, Republic of Korea; 6 Department of Physical Education, Hallym University, Chuncheon, Republic of Korea; 7 Institute of Sports Science, Hallym University, Chuncheon, Republic of Korea

**Keywords:** Cell death in the nervous system, Neurological disorders

## Abstract

Traumatic brain injury (TBI) is a critical condition caused by physical trauma to the head, leading to primary brain edema, hemorrhage, and swelling, along with secondary injuries such as oxidative damage, neuroinflammation, and mitochondrial dysfunction. Astrocytes play a vital role in the astrocyte-neuron lactate shuttle (ANLS), which facilitates the transfer of lactate from astrocytes to neurons as an energy source. This study investigates the role of the pyruvate kinase M2 (PKM2) gene in astrocytes and its impact on neuronal survival following TBI. We hypothesized that deletion of the PKM2 gene in astrocytes would result in increased neuronal death due to impaired lactate supply via the ANLS. Additionally, we hypothesized that lactate administration post-TBI would mitigate neuronal death and alleviate cognitive impairment. To test these hypotheses, we utilized tamoxifen to specifically delete the PKM2 gene in astrocytes of Aldh1l1-Cre^ERT2^; PKM2^f/f^ mice. Following TBI induction, sodium L-lactate was administered, and the mice were sacrificed 24 h later. Our analysis included assessments of neuronal death, microtubule disruption, oxidative damage, and the activity of enzymes associated with the ANLS. The findings confirmed that PKM2 gene deletion in astrocytes exacerbated neuronal death and worsened cognitive impairment. Conversely, lactate administration post-TBI reduced neuronal death and improved cognitive outcomes. These results suggest that lactate administration could serve as a potential therapeutic strategy for preventing and treating neurological damage following TBI.

## Introduction

Traumatic Brain Injury (TBI) stands as a critical public health concern, with roots predominantly in external factors like traffic accidents, sports injuries, and physical violence. Globally, TBI is a leading cause of brain injuries, often resulting in neuronal death and cognitive dysfunction. In the United States alone, about 1.6 million TBIs are reported annually, leading to significant numbers of early treatments and hospitalizations [1, 2]. The impact of TBI varies greatly in severity, ranging from temporary cognitive disruptions to permanent neurological impairments, and in extreme cases, death. Initially, TBI is characterized by primary injuries, such as brain swelling (edema) and internal hemorrhage, setting off a cascade of secondary injuries that include neuronal death, neuro-inflammation, blood-brain barrier disruption, oxidative damage, and the build-up of harmful substances within cells [3, 4].

Understanding the cellular composition of the human brain is crucial in grasping the complexity of TBI. Approximately 90% of brain cells are glial cells (astrocytes, oligodendrocytes, and microglia), while neurons make up about 10% [5]. Under hypoxic conditions induced by TBI or ischemia, these cells undergo significant intracellular regulatory changes [6, 7]. Brain cells metabolize glucose into lactate and pyruvate, while mitochondria engage in oxidative respiration, indicating a shift in metabolic compartmentalization extending beyond mitochondria and cytoplasm [8, 9]. The astrocyte-neuron lactate shuttle (ANLS), increasingly recognized for its importance, functions as a channel for lactate from astrocytes to fuel neurons [10–12]. Astrocytes convert glucose to lactate via lactate dehydrogenase A (LDHA), which is then transported to neurons via monocarboxylate transporters (MCTs) [13, 14].

Pyruvate kinase M2 (PKM2) plays a key role in these cellular energy processes, catalyzing the conversion of phosphoenolpyruvate to pyruvate in glycolysis [15]. PKM2, predominantly found in astrocytes, modulates lactate production, influencing ANLS efficiency [16, 17]. Its interactions with transcription factors like Oct-4, STAT3, and HIF-1α play a part in cellular proliferation and degeneration [11, 16]. Specifically, PKM2’s role in ANLS involves increasing lactate production in astrocytes, thereby enhancing LDHA output, which interacts with HIF-1α for lactate production [18]. In this study, we investigate the impact of astrocyte-specific PKM2 gene deletion using Aldh1l1-Cre^ERT2^; PKM2 floxed and tamoxifen, hypothesizing that this deletion would lead to increased neuronal death in the hippocampus post-TBI due to an imbalance in lactate replenishment via ANLS.

The unique role of PKM2 in response to TBI is critical. As an enzyme crucial in glycolysis, PKM2 converts phosphoenolpyruvate (PEP) to pyruvate, aiding in ATP production [15]. Its existence in two isoforms is particularly important in the brain and proliferative cells like cancer cells. Its ability to adjust metabolic output in response to physiological changes assists not just in energy production, but also in synthesizing biomolecules necessary for cell growth and repair [19]. Deletion of the PKM2 gene could disrupt this metabolic process, resulting in insufficient energy supply in neurons and heightened vulnerability under conditions such as TBI or ischemia [11]. Our study explores the effects of PKM2 gene deletion on neuronal survival following TBI, specifically examining whether PKM2 gene deletion diminishes lactate supply to neurons, exacerbating neuronal death. Using a mouse model with specific PKM2 gene deletion and administering sodium L-lactate post-TBI, we observed that PKM2 gene deletion in astrocytes intensified neuronal damage in the hippocampal region post-ischemia. However, lactate supplementation post-injury significantly mitigated neuronal death, enhancing neuron survival and cognitive functions.

Lactate, once considered merely a byproduct of metabolism, has been recognized as a crucial energy source in the brain, especially after TBI [20, 21]. In such cases, the brain’s energy needs often exceed its supply, resulting in energy deficits [22]. Lactate supplementation thus emerges as a potential alternative energy source for neurons, compensating for the metabolic stress caused by TBI. The role of lactate in this context, and its effectiveness in promoting neuronal survival and recovery post-TBI, are subjects of ongoing research and could significantly advance our understanding of neuronal recovery mechanisms.

In conclusion, our study provides valuable insights into the intricate roles of PKM2 and lactate in neuronal metabolism and survival in the context of TBI. It highlights the potential of targeting metabolic pathways for therapeutic intervention in TBI and similar neurological conditions. As our understanding of these metabolic interactions deepens, we open the door to developing more effective treatment strategies for TBI, potentially improving outcomes for those affected by such injuries.

## Results

### Effects of PKM2 gene deletion in astrocytes following tamoxifen administration

**Fig. 1:**
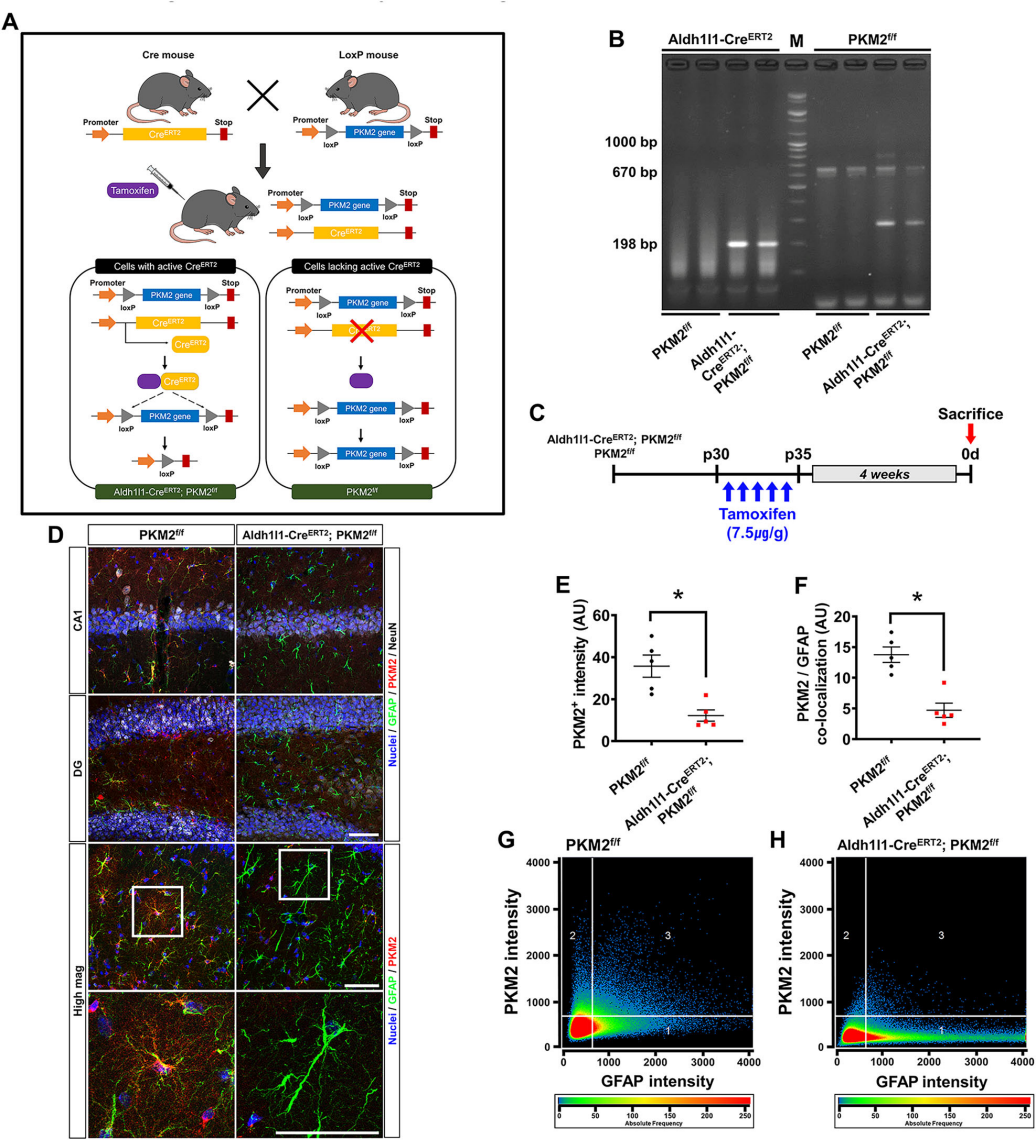
Effects of PKM2 gene deletion in astrocytes following tamoxifen administration. **A** Diagram showing the conditional knockout of the PKM2 gene in the Aldh1l1-Cre^ERT2^; PKM2^f/f^ model following tamoxifen administration. **B** A representative gel PCR genotyping image displaying a 198-bp band specific to the Aldh1l1-Cre^ERT2^ gene and a 670-bp band indicative of the PKM2^f/f^ gene. The 300-bp band observed corresponds to the Cre-mediated recombination product of the floxed PKM2 allele in astrocyte. **C** Outlines the administration protocol of tamoxifen (7.5 μg/g, i.p.) administered once daily for a total of 5 days, with mice sacrificed after 4 weeks. **D** Shows the expression of PKM2 (red), astrocytes (marked by GFAP, green) and live neuron (marked by NeuN, white) in the hippocampus region CA1 and DG and high magnification image. Scale bar represents 50 μm. **E**, **F** Graphical representation of PKM2 intensity and the co-localization of PKM2 and GFAP in the hippocampal region (PKM2^f/f^, n = 5; Aldh1l1-Cre^ERT2^-PKM2^f/f^, n = 5) (Mann–Whitney U test measurement results, PKM2^+^ intensity: z = –2.611, p = 0.008, PKM2/GFAP co-localization: z = –2.611, p = 0.008). **G, H** Scatterplot diagrams illustrating the extent of PKM2 and GFAP co-localization. Data are presented as mean ± SEM. Asterisk (*) indicates a significant difference from the PKM2^f/f^ group in the Aldh1l1-Cre^ERT2^; PKM2^f/f^ group (p < 0.05).

To investigate the effects of Pyruvate Kinase M2 (PKM2) gene deletion in astrocytes following tamoxifen administration, we focused on understanding how conditional knockout of PKM2 affects astrocytic function and potentially influences brain physiology, particularly in the hippocampus. By using the Aldh1l1-Cre^ERT2^; PKM2^f/f^ model for conditional knockout and administering tamoxifen, we aimed to reduce PKM2 expression in astrocytes and then analyze the consequences. The study involved PCR genotyping to confirm gene deletion and immunofluorescence analysis to observe changes in PKM2 expression and its co-localization with Glial Fibrillary Acidic Protein (GFAP), a marker for astrocytes. Fig. 1A illustrates the conditional knockout of the PKM2 gene in Aldh1l1-Cre^ERT2^; PKM2^f/f^ astrocytes following tamoxifen administration. PCR genotyping confirmed the specific gene, with representative bands observed for Aldh1l1-Cre^ERT2^ and PKM2^f/f^ genes. We also observed to the Cre-mediated recombination product of the floxed PKM2 allele in astrocyte, confirming successful conditional deletion. So, we used the Aldh1l1- Cre^ERT2^ primer to check that a band appears at 198-bp (Fig. 1B). Mice were administered with tamoxifen for 5 consecutive days after post-30 days and sacrificed after 4 weeks (Fig. 1C). The immunofluorescence analysis showed PKM2, astrocyte and live neuron expression in hippocampal CA1 and DG region of Aldh1l1-Cre^ERT2^; PKM2^f/f^ and PKM2^f/f^ mice. And It also showed PKM2 and astrocyte expression to high magnification image in hippocampus region (Fig. 1D). The PKM2 expression in astrocyte reduced with significant difference in PKM2 intensity and GFAP co-localization when compared to the PKM2^f/f^ groups (PKM2^+^ intensity reduced by about 66.1%; PKM2f/f: 35.6 ± 5.2, Aldh1l1-Cre^ERT2^; PKM2^f/f^: 12.1 ± 2.6, PKM2/GFAP co-localization reduced by about 65.7%, PKM2^f/f^: 13.7 ± 1.2, Aldh1l1-Cre^ERT2^; PKM2^f/f^: 4.7 ± 1.1) (Fig. 1E, F). PKM2 expression in astrocyte was reduced, confirming the co-localization of PKM2 and GFAP in scatter diagram analysis (Fig. 1G, H).

### Effects of lactate supplementation on scratch wound healing in primary neuron cultures

**Fig. 2:**
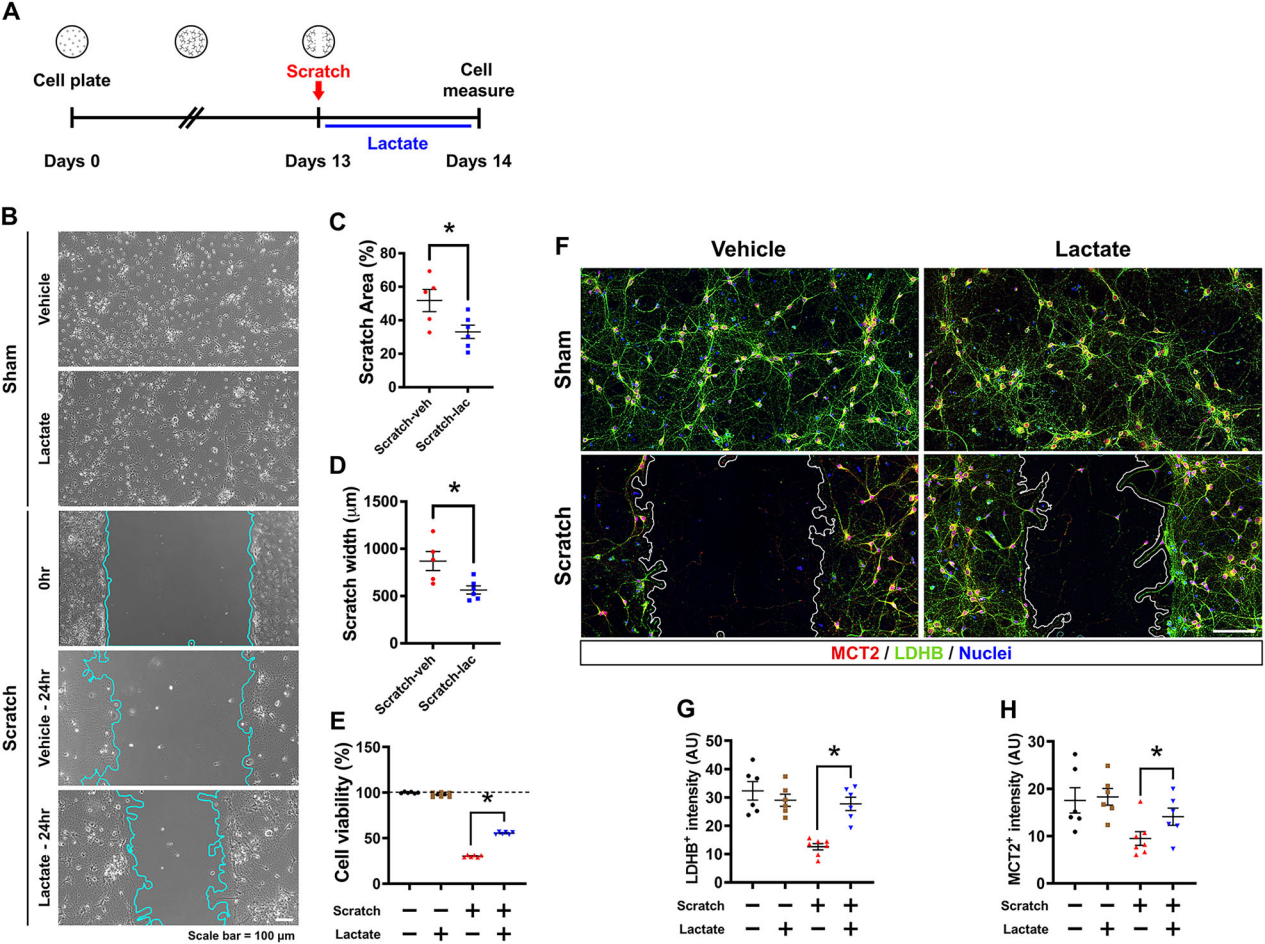
Effects of lactate supplementation on wound healing recovery in primary neuronal cultures. **A** Illustrates the protocol for primary neuron culture, including the timeline for scratch injury and subsequent lactate administration. **B** Images depict wound healing in primary neuron culture following lactate supplementation post-scratch injury. Scale bar = 100 μm. **C**, **D** Graphs present the percentage change in scratch area and the width (μm) in primary neuron cultures post-injury (Scratch-vehicle group, n = 5; Scratch-lactate group, n = 6). (Mann–Whitney U test measurement results, scratch area (%): z = –2.008, p = 0.052, scratch width (μm): z = –2.373, p = 0.017) **E** Graphs shows the percentage change in cell survival in the primary neuron cultures after scratch induction. (Sham-vehicle group, Sham-lactate group, Scratch-vehicle group, Scratch-lactate group; n = 6–8 each). (Bonferroni post hoc test after Kruskal-Wallis test, Cell viability (%): chi square = 19.767, df = 3, p = < 0.001) **F** Shows merged images of MCT2 (red) and LDHB (green) in primary neuron culture following scratch injury. Scale bar = 100 μm. **G, H** Bar graphs displaying the intensity of LDHB and MCT2 in primary neuron cultures post-lactate supplementation (Sham-vehicle, n = 6; Sham-lactate, n = 6; Scratch-vehicle, n = 7; Scratch-lactate, n = 6). Data represent mean ± SEM. An asterisk (*) indicates a significant difference between the Scratch-vehicle and Scratch-lactate groups (p < 0.05) (Bonferroni post hoc test after Kruskal-Wallis test, LDBH^+^ intensity: chi square = 15.135, df = 3, p = 0.002, MCT2^+^ intensity: chi square = 10.575, df = 3, p = 0.014).

The purpose of this study is to investigate the effects of lactate supplementation on wound healing and neuronal recovery in primary neuron cultures after a scratch injury. The study aims to determine whether adding lactate to the culture medium can enhance the healing process in neurons and improve cell viability compared to control conditions without lactate supplementation. Through experiments involving scratch injury in neuron cultures and subsequent treatment with lactate, the researchers assess wound healing, cell viability, and the expression of specific proteins involved in lactate metabolism. The timeline in Fig. 2A shows to process primary neuron cultures for 13 days, and changing the medium in the presence or absence of lactate after scratching, and measurements after 24 hr. The image shows that when each group was compared to the scratch-vehicle group (0 hr), the scratch-lactate group (24 hr) showed reduced scratch injury compared to the scratch-vehicle group (24 hr) (Fig. 2B). The scratch area (%) and width (µm) reduced with significant difference in scratch-lactate group when compare to scratch-vehicle group (Scratch Area (%) reduced by about 36%, Scratch-vehicle: 51.7 ± 6.6, Scratch-lactate: 33 ± 3.9, Scratch width (µm) reduced by 35%, Scratch-vehicle: 870.3 ± 100.7, Scratch-lactate: 564.4 ± 42.7) (Fig. 2C, D). The cell viability was not different in the sham groups, but increased in the scratch-lactate group compared to the scratch-vehicle group in the cell viability (Cell viability increased by 45%, Scratch-vehicle: 30.1 ± 0.5, Scratch-lactate: 55.4 ± 0.5) (Fig. 2E). The fluorescence imaging also showed the expression MCT2 and LDHB in primary neuron culture (Fig. 2F). The MCT2 and LDHB expression increased with significant difference in scratch-lactate group when compare to scratch-vehicle group (LDHB expression increased by about 54%, Scratch-vehicle: 12.5 ± 1.0, Scratch-lactate: 27.7 ± 2.3, MCT2 expression increased by about 32%, Scratch-vehicle: 9.4 ± 1.4, Scratch-lactate: 14 ± 1.8) (Fig. 2G, H). This part of the study is significant for exploring the potential therapeutic uses of lactate.

### Impact of PKM2 gene deletion on neuronal death and ATP levels after TBI

**Fig. 3:**
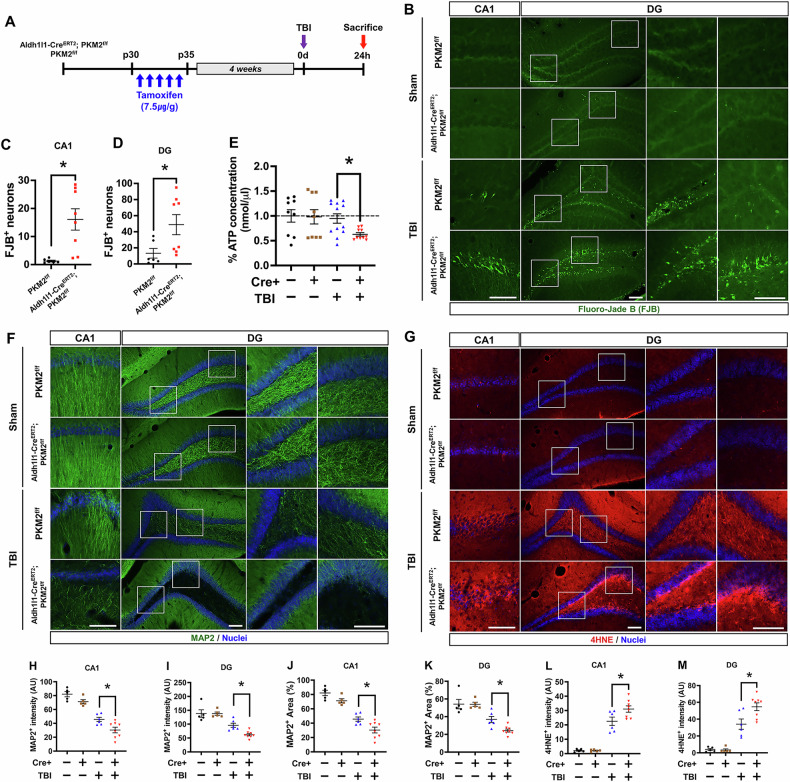
Impact of PKM2 gene deletion on neuronal death, ATP levels, microtubule and reactive oxygen species after TBI. **A** Schematic representation detailing the timeline, including TBI induction and subsequent sacrifice of mice 24 h post-injury. **B** Images display degenerated neurons (stained with FJB, green) in the hippocampal CA1 and DG regions after TBI. Scale bar = 100 μm. **C, D** Graphs compare the number of degenerated neurons in the TBI-PKM2^f/f^ group with the Aldh1l1-Cre^ERT2^; PKM2^f/f^ group. (TBI-PKM2^f/f^ group, n = 6; TBI-Aldh1l1-Cre^ERT2^; PKM2^f/f^ group, n = 8) (Mann-Whitney U test measurement results, DG: z = 2.066, p = 0.043, CA1: z = 2.969, p = 0.001). **E** Graph shows the normalized percentage of ATP concentration to total protein concentration in the TBI-PKM2^f/f^ group compared with the Aldh1l1-Cre^ERT2^; PKM2^f/f^ group. (sham-PKM2^f/f^ group, n = 9; sham-Aldh1l1-Cre^ERT2^; PKM2^f/f^ group, n = 9; TBI-PKM2^f/f^ group, n = 12; TBI-Aldh1l1-Cre^ERT2^; PKM2^f/f^ group, n = 11). Data are mean ± SEM. An asterisk (*) indicates a significant difference from the TBI-PKM2^f/f^ group in the TBI-Aldh1l1-Cre^ERT2^; PKM2^f/f^ group (p < 0.05) (Bonferroni post hoc test after Kruskal-Wallis test, %ATP concentration: chi square = 4.919, df = 3, p = 0.178). **F** Fluorescence image displaying microtubule-associated protein 2 (MAP2, green) staining indicating microtubule disruption in hippocampal CA1 and DG regions 24 h after TBI. Scale bar = 100 µm. **G** Fluorescence image showing reactive oxygen species (ROS) indicated by 4-hydroxynonenal (4HNE, red) staining in hippocampal CA1 and DG regions 24 h after TBI. Scale bar = 100 µm. **H–K** Graphs presenting the intensity and percent area of MAP2 staining in the CA1 and DG regions, illustrating the extent of microtubule disruption. **L, M** Graphs depict the intensity of 4HNE staining in the CA1 and DG regions, highlighting the increase in ROS post-TBI. Includes data from sham-PKM2^f/f^ group (n = 5), sham-Aldh1l1-Cre^ERT2^; PKM2^f/f^ group (n = 5), TBI-PKM2^f/f^ group (n = 6), and TBI-Aldh1l1-Cre^ERT2^; PKM2^f/f^ group (n = 8). Data are presented as mean ± SEM. An asterisk (*) denotes significant differences from the TBI-PKM2^f/f^ to the TBI-Aldh1l1-Cre^ERT2^; PKM2^f/f^ group (p < 0.05) (Bonferroni post hoc test after Kruskal-Wallis test, MAP2; DG: chi square = 19.496, df = 3, p = < 0.001, CA1: chi square = 19.208, df = 3, p = < 0.001, 4HNE; DG: chi square = 18.708, df = 3, p = < 0.001, CA1: chi square = 18.114, df = 3, p = < 0.001).

The study aims to understand how the conditional knockout of PKM2 in astrocytes influences the degenerating neuron in hippocampal region of CA1 and dentate gyrus (DG), and the availability of ATP in the hippocampus following TBI. Through fluorescent imaging and quantitative analysis, the study assesses the extent of neuronal degeneration and compares ATP concentrations between the knockout group and controls. The timeline in Fig. 3A shows that both the TBI-induced PKM2 knockout and control groups were sacrificed after 24 h. The fluorescence image was observed degenerated neurons in the hippocampal regions of CA1 and DG with Fluoro-Jade B staining (Fig. 3B). Quantitative analysis confirmed increased with significantly difference FJB-positive neurons in TBI-induced Alhd1l1-Cre^ERT2^; PKM2^f/f^ group when compare to TBI-induced PKM2^f/f^ group (CA1 region increased about by 73%, TBI-PKM2^f/f^: 13.2 ± 5.9, TBI-Aldh1l1-Cre^ERT2^; PKM2^f/f^: 48.8 ± 12.4, DG region increased about by 92%, TBI-PKM2^f/f^: 1.2 ± 0.3, TBI-Aldh1l1-Cre^ERT2^; PKM2^f/f^: 16 ± 3.8) (Fig. 3C, D). Additionally, ATP concentration was significantly lower in the TBI-Aldh1l1-Cre^ERT2^; PKM2^f/f^ group compare to TBI-PKM2^f/f^ group (%ATP concentration reduced about by 33%, TBI-PKM2^f/f^: 0.9 ± 0.09, TBI-Aldh1l1-Cre^ERT2^; PKM2^f/f^: 0.6 ± 0.06) (Fig. 3E).

### Conditional PKM2 gene deletion in astrocytes aggravates microtubule disruption and reactive oxygen species post-TBI

We investigated how the deletion of the PKM2 gene impacts microtubule integrity and reactive oxygen species (ROS) production in the hippocampal region following TBI. Examine the structural integrity of microtubules in neurons, as the stability of these cellular structures is vital for neuron function and survival. Microtubule disruption is assessed using microtubule-associated protein 2 (MAP2) staining in the hippocampal CA1 and DG regions after TBI (Fig. 3F). ROS are byproducts of cellular metabolism, and their excess production can cause oxidative stress, damaging cells and tissues. We uses 4-hydroxynonenal (4HNE) staining as a marker for ROS levels, particularly focusing on whether PKM2 gene deletion exacerbates ROS production in the hippocampal region of TBI (Fig. 3G). MAP2 intensity and area percent reduced with significantly difference in TBI-Aldh1l1-Cre^ERT2^; PKM2^f/f^ group when compare to TBI-PKM2^f/f^ group (MAP2 intensity reduced about by 33% in CA1, TBI-PKM2^f/f^: 45.6 ± 3.0, TBI-Aldh1l1-Cre^ERT2^; PKM2^f/f^: 30.4 ± 4, by 35% in DG, TBI-PKM2^f/f^: 95.7 ± 7.6, TBI-Aldh1l1-Cre^ERT2^; PKM2^f/f^: 61.8 ± 4.2, MAP2 area (%) reduced about by 33% in CA1, TBI-PKM2^f/f^: 17.8 ± 1.1, TBI-Aldh1l1-Cre^ERT2^; PKM2^f/f^: 11.9 ± 1.5, by 34% in DG, TBI-PKM2^f/f^: 36.8 ± 3.3, TBI-Aldh1l1-Cre^ERT2^; PKM2^f/f^: 24.2 ± 1.6) (Fig. 3H–K). Also, we confirmed that ROS levels, as measured by 4HNE staining, were significantly enhanced in the TBI-Aldh1l1-Cre^ERT2^; PKM2^f/f^ groups compare to TBI-PKM2^f/f^ group (4HNE intensity increased about by 27% in CA1, TBI-PKM2^f/f^: 22.5 ± 2.9, TBI-Aldh1l1-Cre^ERT2^; PKM2^f/f^: 31 ± 2.2, by 37% in DG, TBI-PKM2^f/f^: 34 ± 6.1, TBI-Aldh1l1-Cre^ERT2^; PKM2^f/f^: 54.7 ± 4.5) (Fig. 3L, M).

### Effects of PKM2 gene deletion in astrocytes on the lactate pathway after TBI

**Fig. 4:**
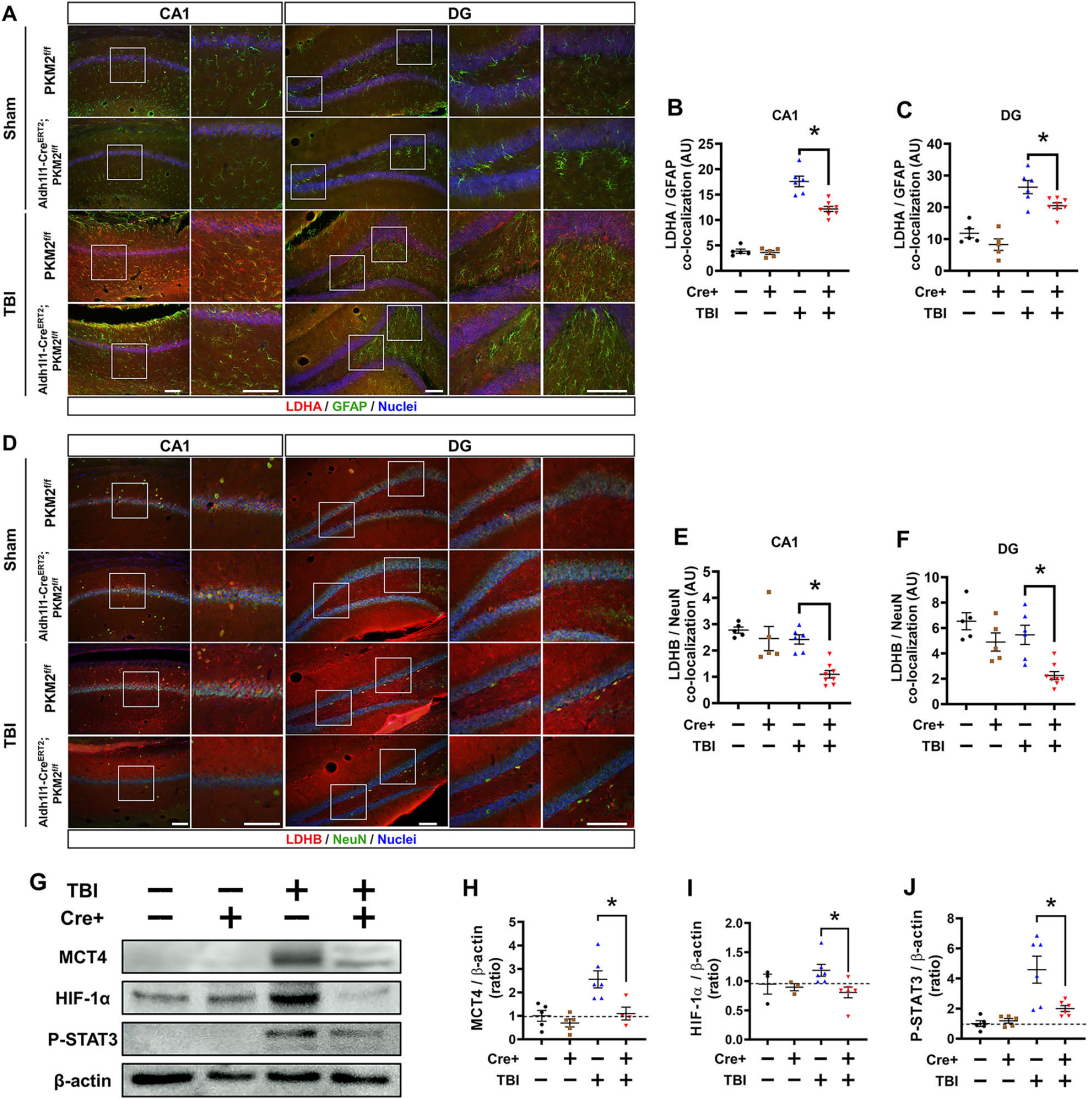
Effects of PKM2 gene deletion in astrocytes on lactate pathway after TBI. **A** Fluorescent image showing lactate dehydrogenase A (LDHA, red) and astrocyte marker (GFAP, green) merged in the CA1 and DG regions after TBI. Scale bar = 100 µm. **B, C** Bar graphs depicting the intensity of co-localization between LDHA and GFAP in the CA1 and DG regions. Includes data from sham-PKM2^f/f^ group (n = 5), sham-Aldh1l1-Cre^ERT2^; PKM2^f/f^ group (n = 5), TBI-PKM2^f/f^ group (n = 6), and TBI-Aldh1l1-Cre^ERT2^; PKM2^f/f^ group (n = 8) (Bonferroni post hoc test after Kruskal-Wallis test, DG: chi square = 18.487, df = 3, p = < 0.001, CA1: chi square = 20.162, df = 3, p = < 0.001). **D** Fluorescent image displaying lactate dehydrogenase B (LDHB, red) and neuronal marker (NeuN, green) in the CA1 and DG regions following TBI. Scale bar = 100 μm. **E, F** Bar graphs showing the co-localization intensity of LDHB and NeuN in the CA1 and DG regions. Data from sham-PKM2^f/f^ group (n = 5), sham-Aldh1l1-Cre^ERT2^; PKM2^f/f^ group (n = 5), TBI-PKM2^f/f^ group (n = 6), and TBI-Aldh1l1-Cre^ERT2^; PKM2^f/f^ group (n = 8). Data are mean ± SEM. An asterisk (*) indicates significant differences (p < 0.05) between the TBI-PKM2^f/f^ and TBI-Aldh1l1-Cre^ERT2^; PKM2^f/f^ groups (Bonferroni post hoc test after Kruskal-Wallis test, CA1: chi square = 15.881, df = 3, p = 0.001, DG: chi square = 14.225, df = 3, p = 0.003). **G** Displays the expression levels of monocarboxylate transporter 4 (MCT4), phosphorylated signal transducer and activator of transcription 3 (P-STAT3) and hypoxia inducible factor 1 – alpha (HIF-1α) in the hippocampus post-TBI. **H–J** Graphs illustrate the relative levels of MCT4, P-STAT3, HIF-1α and normalized to β-actin, as determined by western blot analysis. Sample groups: each sham groups (n = 5 each), TBI groups (n = 6 for PKM2^f/f^, n = 4-6 for Aldh1l1-Cre^ERT2^; PKM2^f/f^). Date are means ± SEM are provided, with an asterisk (*) indicating significant differences (p < 0.05) between the TBI-PKM2^f/f^ and TBI-Aldh1l1-Cre^ERT2^; PKM2^f/f^ groups (Bonferroni post hoc test after Kruskal-Wallis test, MCT4/β-actin: chi square = 11.858, df = 3, p = 0.008, HIF-1α/β-actin: chi square = 8.333, df = 3, p = 0.040, P-STAT3/β-actin: chi square = 15.983, df = 3, p = 0.001).

The study aims to determine how the conditional knockout of the PKM2 gene impacts the expression of the lactate dehydrogenase A and B (LDHA, LDHB) in astrocyte and monocarboxylate transporter 4 (MCT4) of lactate transporter in the brain post-TBI. Through fluorescent imaging, the study assesses the levels of LDHA and GFAP co-localization in the hippocampal region CA1 and DG after TBI (Fig. 4A). LDHA and GFAP co-localization reduced with significantly difference in the TBI-Aldh1l1-Cre^ERT2^; PKM2^f/f^ group when compare to TBI-PKM2^f/f^ group (LDHA and GFAP co-localization reduced about by 31% in CA1, TBI-PKM2^f/f^: 17.6 ± 1, TBI-Aldh1l1-Cre^ERT2^; PKM2^f/f^: 12.1 ± 0.5, 22% in DG, TBI-PKM2^f/f^: 26.3 ± 2, TBI-Aldh1l1-Cre^ERT2^; PKM2^f/f^: 20.5 ± 0.9) (Fig. 4B, C). This co-localization image assess the levels of LDHB and NeuN in the hippocampal regions after TBI (Fig. 4D). LDHB and NeuN co-localization reduced with significantly difference in the TBI-Aldh1l1-Cre^ERT2^; PKM2^f/f^ group when compare to TBI-PKM2^f/f^ group (LDHB and NeuN co-localization reduced about by 58% in CA1, TBI-PKM2^f/f^: 2.4 ± 0.1, TBI-Aldh1l1-Cre^ERT2^; PKM2^f/f^: 1.0 ± 0.1, 59% in DG, TBI-PKM2^f/f^: 5.4 ± 0.7, TBI-Aldh1l1-Cre^ERT2^; PKM2^f/f^: 2.2 ± 0.3) (Fig. 4E, F). Through western blot analysis, the study confirms the expression of MCT4, phosphorylate signal transducer and activator of transcription 3 (P-STAT3) and hypoxia inducible factor 1-alpha (HIF-1α) in the hippocampal region after TBI (Fig. 4G). The MCT4, P-STAT3 and HIF-1α protein levels reduced with significantly difference TBI-Aldh1l1-Cre^ERT2^; PKM2^f/f^ group when compare to TBI-PKM2^f/f^ group (MCT4/β-actin reduced about by 58%, TBI-PKM2^f/f^: 2.5 ± 0.3, TBI-Aldh1l1-Cre^ERT2^; PKM2^f/f^: 1.0 ± 0.2, P-STAT3/β-actin reduced about by 62%, TBI-PKM2f/f: 4.5 ± 0.8, TBI-Aldh1l1-Cre^ERT2^; PKM2^f/f^: 2.0 ± 0.2, HIF-1α/ β-actin reduced about by 32%, TBI-PKM2^f/f^: 1.2 ± 0.1, TBI-Aldh1l1-Cre^ERT2^; PKM2^f/f^: 0.8 ± 0.09) (Fig. 4H–J). The reduced levels of these lactate pathway factors in the knockout group suggest alterations in lactate production and transport, which are critical for energy metabolism from astrocytes to neurons, particularly under stress conditions like TBI.

### Lactate administration mitigates TBI-induced neuronal death in PKM2 gene deletion mice

**Fig. 5:**
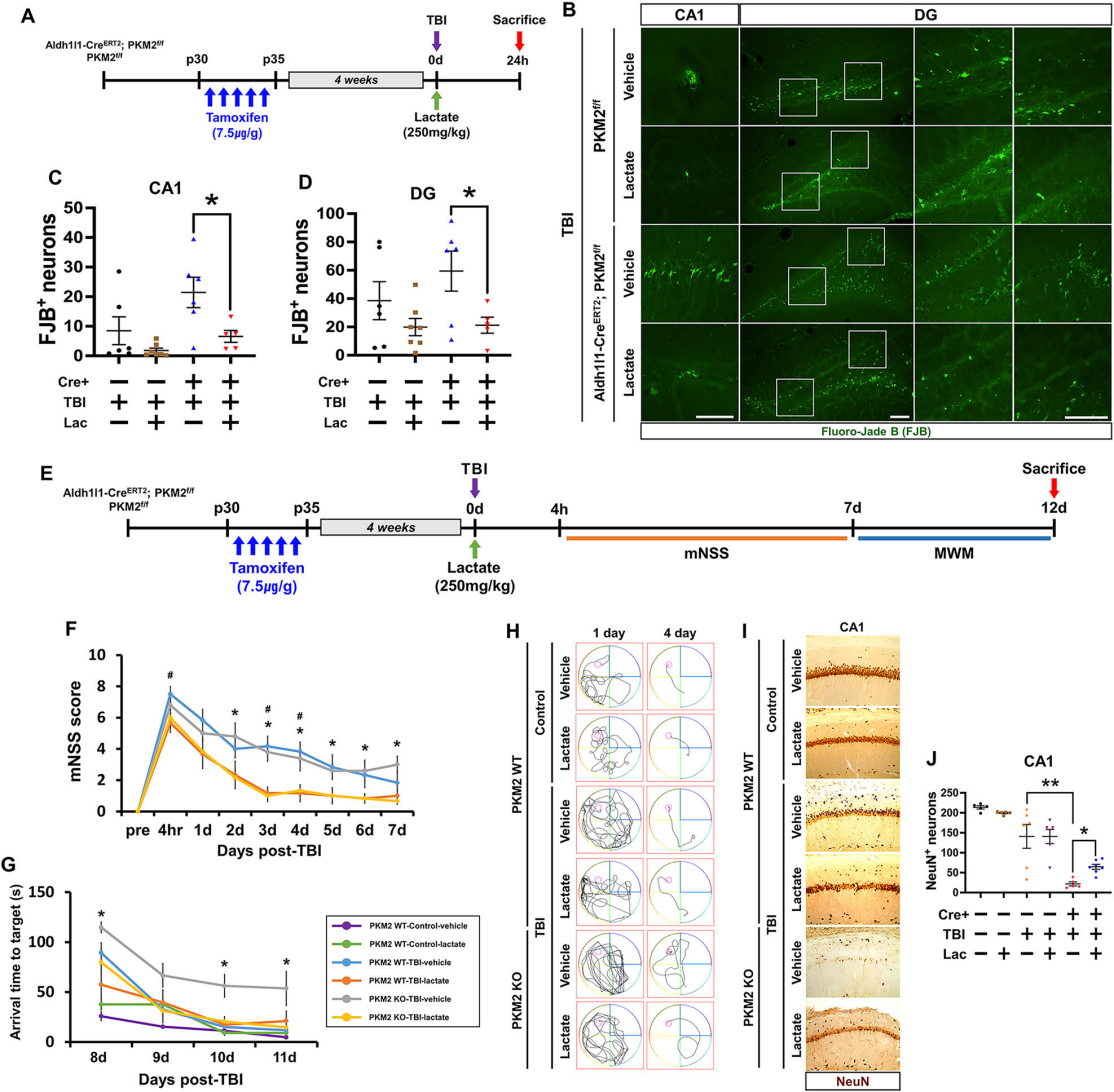
Effects of PKM2 gene deletion in astrocytes on neuronal death and cognitive function following TBI. **A** Schematic representation detailing the timeline. **B** Fluorescence image displaying degenerated neurons (stained with FJB, green) in the hippocampal CA1 and DG regions after TBI. Scale bar = 100 μm. **C, D** Graphs compare the number of degenerated neurons in the TBI-PKM2^f/f^-vehicle (n = 6), TBI-PKM2^f/f^-lactate (n = 7), TBI-Aldh1l1-Cre^ERT2^; PKM2^f/f^-vehicle (n = 6), and TBI-Aldh1l1-Cre^ERT2^; PKM2^f/f^-lactate (n = 5) groups. Data are presented as means ± SEM, with an asterisk (*) indicating significant differences (p < 0.05) between the TBI-Aldh1l1-Cre^ERT2^; PKM2^f/f^-vehicle and TBI-Aldh1l1-Cre^ERT2^; PKM2^f/f^-lactate groups (Bonferroni post hoc test after Kruskal-Wallis test, DG: chi-square = 4.906, df = 3, p = 0.179; CA1: chi-square = 11.432, df = 3, p = 0.010). **E** The schematic timeline shows the total duration of the modified neurological severity score (mNSS) and Morris water maze (MWM) test after TBI induction. **F** Graphs represent mNSS testing in each of the vehicle and lactate groups on 7 consecutive days after TBI induction in WT and KO mice. **G** Graphs representing arrival time to target to MWM tests performed in WT and KO mice, PKM2 WT-controls, on 4 consecutive days after TBI induction in each vehicle and lactate group (n = 5–7 for each group). Data are presented as means ± SEM, with an asterisk (*, ^#^) indicating significant differences (p < 0.05) between the PKM2 WT-TBI-lactate and PKM2 WT-TBI-vehicle groups, or the PKM2 KO-TBI-lactate and PKM2 KO-TBI-vehicle groups (repeated measures test followed by ANOVA; mNSS test: PKM2 KO group, time: F = 28.220, p < 0.001; timegroup: F = 28.220, p < 0.001; group: F = 13.810, p = 0.005; MWM test: PKM2 KO group, time: F = 28.306, p < 0.001; time*group: F = 1.417, p = 0.248; group: F = 10.825, p = 0.009). **H** The average trace of the water maze test for 1 day and 4 days for each of the 6 groups. **I** Image displaying surviving neurons (stained with NeuN) in the hippocampal CA1 regions after TBI. Scale bar = 100 μm. **J** Graphs comparing the number of surviving neurons in each vehicle and lactate group (n = 5–7 for each group). Data are presented as means ± SEM, with asterisks (*, **) indicating significant differences (p < 0.05) between the TBI-PKM2 WT-vehicle, TBI-PKM2 KO-vehicle, and TBI-PKM2 KO-lactate groups (Mann–Whitney U test measurement results; **: z = –2.556, p = 0.009, *: z = 2.680, p = 0.005).

This study aims to investigate the effects of lactate administration after TBI in PKM2 gene deletion mice. Figure 5A shows that both PKM2 WT and KO groups were sacrificed 24 h after lactate injection, which was administered immediately following TBI induction. The fluorescence images in Fig. 5B reveal degenerating neurons in the hippocampal regions of CA1 and DG, identified using Fluoro-Jade B staining. Our findings indicate that neuronal death was reduced in TBI-induced PKM2 WT and KO groups after lactate administration in the hippocampal regions of CA1 and dentate gyrus (DG). Quantitative analysis confirmed a significant reduction in FJB-positive neurons in the TBI-induced Aldh1l1-Cre^ERT2^; PKM2^f/f^-lactate group compared to the TBI-induced Aldh1l1-Cre^ERT2^; PKM2^f/f^ group. Specifically, the CA1 region showed a reduction of about 69% (TBI-Aldh1l1-Cre^ERT2^; PKM2^f/f^: 21.4 ± 5.1, TBI-Aldh1l1-Cre^ERT2^; PKM2^f/f^-lactate: 6.5 ± 1.9) and the DG region showed a reduction of about 64% (TBI-Aldh1l1-Cre^ERT2^; PKM2^f/f^: 59.4 ± 14.1, TBI-Aldh1l1-Cre^ERT2^; PKM2^f/f^-lactate: 21.1 ± 5.6) (Fig. 5C, D).

### Effects of PKM2 gene deletion in astrocytes on neurobehavioral and cognitive functions of lactate administration after TBI induction

To determine neurobehavioral and cognitive function, we were performed a modified neurological severity score (mNSS) and Morris water maze (MWM) test. The timeline in Fig. 5E shows the mNSS test being performed 4 h after administration of lactate for induced TBI, then once a day 1, 2, 3, 4, 5, 6, and 7, and the MWM test being performed once a day for days 8, 9, 10, 11 and 12. Graphs represent mNSS and MWM testing for TBI-induced vehicle and lactate groups in each PKM2 WT and KO over 7 days. The mNSS test was significantly reduced in the PKM2 WT-TBI-lactate group at 4 h, 3 and 4 days and the PKM2 KO-TBI-lactate group at 2, 3, 4, 5, 6, and 7 days compared to the respective TBI-vehicle groups. In addition, the PKM2 KO-TBI-lactate groups had a significant decreases in MWM testing compared to the PKM2 KO-TBI-vehicle groups on days 8, 10, and 11 (Fig. 5F, G). Looking at the average tracking history for 1 and 4 days, we see that 4 days is much faster than 1 day for each group. However, it can be seen that the PKM2 KO-TBI-vehicle group took longer than the other groups to reach the hidden platform on Day 4 (Fig. 5H). The images in Fig. 5I also shows surviving neurons in the hippocampal region of CA1 identified using NeuN stating. Our findings showed that surviving neurons were reduced in the TBI-induced PKM2 KO group compare to the TBI-induced PKM2 WT group. Also, the number of surviving neurons was significantly increased in the TBI-induced PKM2 KO-lactate group than in the TBI-induced PKM2 KO-vehicle group. Quantitative analysis confirmed a significant reduction in NeuN-positive neurons in the TBI-induced PKM2 KO-vehicle group compared to the TBI-induced PKM2 WT-vehicle group, and a significant increase in the TBI-induced PKM2 KO-lactate group compared to the TBI-induced PKM2 KO-vehicle group. Specifically, the CA1 region showed a reduction of about 84%, and increase of about 66% (TBI-PKM2 WT-vehicle: 141 ± 29, TBI-PKM2 KO-vehicle: 21.8 ± 4.9, TBI-PKM2 KO-lactate: 64.6 ± 6.2) (Fig. 5J).

## Discussion

**Fig. 6:**
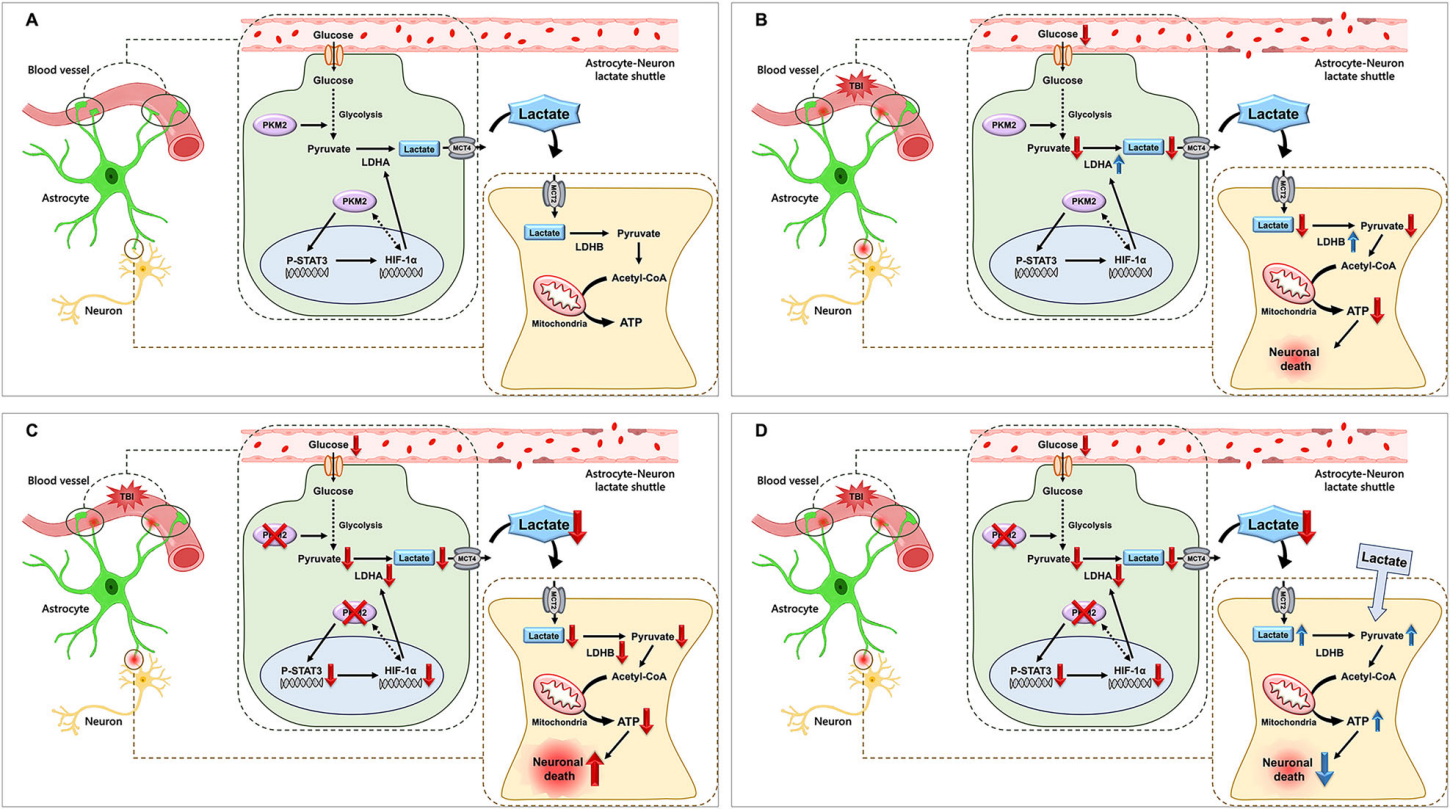
The hypothesis illustration demonstrates neuronal death via the astrocyte-neuron lactate shuttle (ANLS) mechanism following traumatic brain injury (TBI) in the context of PKM2 gene deletion. **A** Under normal conditions, neurons convert lactate delivered through the ANLS into pyruvate, which is then used to produce ATP. **B** However, when TBI occurs, the amount of glucose received by astrocytes from blood vessels decreases, resulting in reduced production of pyruvate and lactate. Consequently, neurons receive less lactate through the ANLS, leading to decreased ATP production and ultimately neuronal death. **C** Following PKM2 gene deletion after TBI, pyruvate production is further reduced due to glycolysis imbalance, and PKM2 does not bind to HIF-1α, leading to decreased LDHA expression. Consequently, reduced lactate production via the ANLS leads to decreased ATP production in neurons, resulting in increased neuronal death. **D** Lactate supplementation reduces neuronal death by increasing energy production in neurons, rather than relying on lactate delivery via the ANLS.

The present study suggests important insights into the role of pyruvate kinase m2 (PKM2) in astrocytes, and its broader implications about lactate pathway in the context of traumatic brain injury (TBI). Our finding reveal significant alterations in astrocyte functioning, neuronal viability and lactate pathway factors after PKM2 gene deletion following TBI. Under normal conditions, astrocytes deliver lactate to neurons as an energy source through the astrocyte-neuronal lactate shuttle (ANLS). PKM2 is involved in this process and in the expression of LDHA, which plays a crucial role in the production of lactate in astrocytes [5, 23]. Our study demonstrates a significant reduction in PKM2 expression in astrocytes following tamoxifen-induced conditional knockout (KO), particularly affecting the hippocampus, a region crucial for memory and learning. This finding is consistent with previous studies that have emphasized the lactate shuttle in astrocytes in brain function and neuroprotection [5, 11, 12] (Fig. 6).

To confirm its importance via the ANLS, we performed conditional PKM2 KO using Aldh1l1-Cre^ERT2^; PKM2^f/f^ and tamoxifen. We found by co-localization of PKM2 and GFAP that PKM2 expression in astrocytes after tamoxifen administration was reduced in the Aldh1l1-Cre^ERT2^; PKM2^f/f^ group. This confirmed that the Aldh1l1-Cre^ERT2^ gene reduces astrocyte-specific PKM2 expression. We observed a significant reduction in the astrocytic PKM2 KO group, which aligns with previous studies showing that even modest changes in metabolic coupling can substantially affect neuronal survival after injury. In previous study, the neuroprotective effects of lactate on neurons have been widely discussed in the literature [24–26]. Based on this, to determine the effects of lactate on scratched neurons, primary neuronal cultures were scratch-induced and then treated with lactate for 24 hr to confirm the extent of wounding. We found that administering lactate after scratching reduced the extent of the wound and increased cell viability. We used EZ-cytox to confirm cell viability, which is determined by an increase in absorbance due to increased mitochondrial dehydrogenase activity in survival cell. We replaced the cell culture medium of all groups before using the EZ-cytox solution to avoid affecting lactate in the medium [27, 28]. As a result, the sham group showed no difference in cell viability, but the group administered lactate in the scratch group increase compare to the scratch group. Also, LDHB and MCT2 expression increased in the lactate administration group after scratching. Through this, we found that administration of lactate after scratching increased cell viability due to increased mitochondrial activity that converts lactate to pyruvate.

To determine the previous experiment to in vivo, we deleted the astrocyte-specific PKM2 gene using Aldh1l1-Cre^ERT2^; PKM2^f/f^ and induced TBI. We determined that deleting the PKM2 gene in astrocytes after TBI increases neurodegeneration and decreases ATP levels. These results demonstrated that the metabolic coupling of astrocytes and neurons plays an important role in brain injury scenarios. Also, in the absence of PKM2 after TBI, we found that microtubule disruption was exacerbated and ROS levels were elevated, suggesting that PKM2 plays a role in maintaining cellular stability and counteracting oxidative stress. This extends existing research that explored the impact of astrocyte function on the structural integrity and stress resistance of neurons [29, 30]. These finding suggest that deletion of PKM2 after TBI increases neuronal death due to a decrease in ATP by reducing lactate replenishment to neurons by an unbalanced ANLS. Also, our observations suggest a potential breakdown in the energy supply chain crucial for neuronal survival during stress, supporting the hypothesis that astrocyte-neuron metabolic interplay is a key component of the brain’s response to injury [10, 31].

Our findings also confirmed the crucial role of PKM2 in regulating the lactate production pathway in astrocytes, essential for neuronal energy metabolism, particularly under pathological conditions such as TBI. We confirmed that the reduced levels of LDHA, MCT4, P-STAT3 and HIF-1α following deletion of the PKM2 gene in astrocyte post-TBI suggest a disruption in lactate metabolism that could lead to an energy crisis in neurons. In a previous study, PKM2 is known to increase LDHA expression by binding to the transcription factor of HIF-1α. PKM2 also phosphorylates STAT3, which increases the expression of HIF-1α [32, 33]. To summarize, P-STAT3 is increased to produce HIF-1α, which in turn produces PKM2 or combines with PKM2 to increase LDHA. Through this interaction, astrocytes increase factors associated with PKM2, leading to increased lactate transfer to neurons after TBI. However, the deletion of PKM2 results in decreased P-STAT3, LDHA and HIF-1α, and decreased conversion of pyruvate to lactate. Also, the deletion of PKM2 also results in reduced MCT4, which acts as a lactate transporter in astrocytes. In addition, we confirmed that LDHB levels were reduced in neurons of the PKM2 deletion groups after TBI. Through this result, we found that deleting PKM2 reduced the conversion of lactate to pyruvate after TBI. These findings suggest that deleting PKM2 reduces the production and transfer of lactate in astrocytes after TBI and decreases lactate-induced energy production in neurons, leading to increased neuronal death. These results contribute to the understanding of lactate’s pivotal role in the ANLS, an area receiving increasing attention in brain metabolism research [5, 10, 34]. Also, understanding the molecular pathways through which PKM2 in astrocytes influences these processes could reveal new therapeutic targets for protecting neurons against TBI-induced damage.

Our observations regarding the beneficial effects of lactate supplementation in primary neuronal cultures after injury, as shown in Fig. 2, are particularly promising. This is consistent with previous evidence suggesting that lactate is not only a metabolic substrate, but also a signaling molecule with neuroprotective properties [11, 21, 35]. To determine the in vivo neuroprotective effects of lactate supplementation after TBI, PKM2 gene KO were administrated lactate after inducing TBI. As a result, we found that neuronal death induced by TBI was reduced in both the PKM2 KO and WT lactate groups, but with a significant difference in the PKM2 KO group. Through this, we suggest that lactate supplementation after TBI reduces neuronal death by providing lactate that could not be supplied through ANLS, thereby replenishing neurons with an energy source. In addition, we measured neurobehavioral and cognitive function using the modified neurological severity score (mNSS) and Morris water maze (MWM) after TBI-induced lactate administration. The mNSS and MWM tests were significantly decreased in the PKM2 KO-TBI lactate group when compared to the PKM2 KO-TBI vehicle group. However, the PKM2 WT-TBI lactate group was only significantly reduced at 4 hr, 3, and 4 days. The results showed that lactate supplementation after TBI resulted in faster recovery of neurobehavioral and cognitive function in the PKM2 KO group. However, we found no significant difference in the PKM2 WT group. We also found that the number of neurons that survived after behavioral testing was significantly increased in the TBI-induced PKM2 KO-lactate group compared to the TBI-induced PKM2 KO-vehicle group. This suggests that lactate supplementation after TBI can restore early neurobehavioral, cognitive function and surviving neurons by delivering energy to replace unbalanced ANLS. This raises the potential of lactate supplementation as a therapeutic strategy in conditions of brain injury and neurodegeneration, an area that warrants further exploration.

In summary, this study highlights the multifaceted role of PKM2 in astrocyte metabolism, neuronal support, and the brain’s response to TBI. By identifying changes in the lactate pathway after TBI following PKM2 gene KO, this research contributes to a broader understanding of the metabolic alterations in the brain post-TBI. These findings may inform potential therapeutic strategies targeting metabolic pathways for TBI treatment. Future studies should delve deeper into the molecular mechanisms underlying these observations and explore the therapeutic potential of targeting these pathways.

## Materials and methods

### Ethics statement and animal care management

All experimental procedures involving animals in this study adhered to the ethical guidelines and were approved by the Hallym University Animal Research Committee (HUARC, approval number: Hallym 2022-68, date: 20 February 2023). The mice utilized in the experiments were C57BL/6J strain, aged 2–3 months, and specifically adult males. They were bred and housed in the Hallym University of Medicine facility. The environmental conditions for raising the mice were consistent with those described in a previous study [11]. To minimize discomfort and pain during the experimental procedures, mice were anesthetized with isoflurane at concentrations of 2–3%. This study also adheres to the guidelines outlined in ARRIVE (Animals in Research: Reporting In Vivo Experiments) to ensure high standards of reporting and animal welfare [36].

### PCR genotyping

DNA for PCR genotyping was extracted from mouse toe samples using a DNA extraction kit provided by Bioneer Inc., Daejeon, Korea. Prior to conducting the experiments, PCR genotyping was verified using specific primers: Aldh1l1-Cre^ERT2^ forward (5’-CTTCAACAGGTGCCTTCCA-3’), Aldh1l1-Cre^ERT2^ reverse (5’-GGCAAACGGACAGAAGCA-3’), PKM2^f/f^ forward (5’-CCTTCAGGAAGACAGCCAAG-3’), and PKM2^f/f^ reverse (5’-AGTGCTGCCTGGAATCCTCT-3’). The expected band sizes for the Aldh1l1-Cre^ERT2^ and PKM2^f/f^ genes were 198 bp and 680 bp, respectively. The PCR products were visualized on a 2% agarose gel using ethidium bromide staining to confirm the band sizes.

### Tamoxifen and lactate injection

Four-week-old mice received tamoxifen injections (75 mg/kg, intraperitoneally, Sigma-Aldrich Co., St. Louis, MO, USA), dissolved in corn oil (20 mg/ml), for five consecutive days. The TBI procedure was performed 4–5 weeks post-tamoxifen injection, at which point the mice weighed at least 20 g.

For post-TBI experiments, we administered sodium l-lactate (250 mg/kg, intraperitoneally, Sigma-Aldrich Co., St. Louis, MO, USA) dissolved in 0.9% normal saline. The control (vehicle) groups were injected with only 0.9% normal saline, without sodium L-lactate.

### In vivo methods for traumatic brain injury

The Controlled Cortical Impact model was employed for TBI induction, as described by Choi et al. [37]. Mice were anesthetized using a 3% isoflurane mixture (VetEquip, Livermore, CA, USA) with a 70:30 nitrous oxide: oxygen ratio and then securely positioned in a stereotaxic apparatus (David Kopf Instruments, Tujunga, CA, USA). Following anesthesia, a craniotomy was performed to expose a 3 mm diameter area of the cranial skull, located 1.0 mm lateral to the midline and 2.0 mm posterior to the bregma. TBI was induced using a CCI device (Leica Impact One; Leica Biosystems, Nussloch, Germany), delivering an impact with a 2 mm flat tip at a velocity of 5 m/s and depth of 1.2 mm on the brain surface. Post-TBI, the mice were allowed to recover in a homeothermic incubator maintained at 36.5–37.5 °C (Harvard Bioscience, Holliston, MA, USA). Pathological outcomes were evaluated 24 hr and 2 weeks post-TBI. Mice in the sham groups underwent only the craniotomy procedure.

### Preparation of brain tissue samples

Mice were euthanized for tissue collection at 24 hr and 2 weeks following TBI induction, using urethane anesthesia (1.5 g/kg, intraperitoneally). Post-anesthesia, mice underwent perfusion with 0.9% saline to remove blood, followed by fixation with 4% paraformaldehyde (PFA). The brains were then extracted, further fixed, and stored in 30% sucrose solution until they were fully submerged. For tissue sectioning, the sucrose-infused brains were cryosectioned at a thickness of 30 µm using a cryostat microtome (CM1850, Leica, Wetzlar, Germany).

### Primary neuron culture

At embryonic day 18 (E18), pregnant female mice were anesthetized with 2–3% isoflurane (70% nitrous oxide mixed with 30% oxygen) and embryonic hippocampi were harvest. E18 hippocampi after dissociation with TrypLE (without phenol red, Gibco, Denmark) were cultured in neurobasal medium containing B-27 (Thermo Fisher, Waltham, MA, USA), GlutaMAX (Gibco, Denmark) and penicillin. Isolated hippocampal neurons were plated a concentration of 5 × 10^5^ each in a 24-well plate coated with a poly-L-lysine solution (Sigma-Aldrich, St. Louis, MO, USA). And then, the cultured neurons were maintained at 37.5 °C in a CO_2_ incubator for 2 weeks to allow for proper maturation. After 2 weeks, the mature neurons were fixed for 15 min using 4% paraformaldehyde (PFA), and stored in a 4 °C incubator.

### In vitro methods for scratch model

On the last day of primary neuronal maturation, we used a 200 µl tip to scratch them. Each scratch was given 5 horizontally and 5 vertically at regular intervals in a 24-well plate. After scratching, the neurobasal medium was replaced with regular neurobasal medium with or without lactate. And, the scratched neurons were maintained at 37.5 °C temperature in a CO_2_ incubator for 24 hr. After 24 hr, the cultured neurons were fixed with 4% paraformaldehyde and observed under a microscope. Through scratch wound healing assay, the scratch Area (%) and width (µm) were measured using Image J software (NIH, Bethesda, Rockville, MD, USA) [38].

### Cell viability assay protocol

After scratch-induced, the cultured primary neurons were measured using EZ-cytox solution (cell viability assay kit, DoGenbio CO., Korea). For the EZ-cytox solution, we added 10 µl of solution per 100 µl of medium in each well. Absorbance was measured and analyzed using a microplate reader (Spectramax, Molecular Devices CO., San Jose, CA, USA) at 450 nm.

### ATP assay protocol

To assess ATP levels, we utilized the ATP assay kit (ab83355, Sigma-Aldrich Co., St. Louis, MO, USA). The procedure began with perfusing mice with cold saline, followed by the extraction and cold PBS wash of brain tissue. The hippocampus was sonicated with the addition of 100 µl ATP assay buffer for homogenization. The homogenized tissue was then centrifuged at 13,000 × *g* for 5 min at 4 °C, and the supernatant was collected. To remove enzymes, we used the deproteinizing sample preparation kit—TCA (ab204708, Sigma-Aldrich Co., St. Louis, MO, USA). A 15 µl volume of cold trichloroacetic acid solution/TCA was added to 150 µl of each sample and incubated on ice for 15 min. Following this, the samples were centrifuged at 12,000 × *g* for 5 min, and the supernatant was collected again. Each sample then received 10 µl of cold neutralization buffer, and the mixture was incubated on ice for an additional 5 min. For the assay, both the sample and standard buffer were transferred to a 96-well plate. The ATP levels were quantified by measuring absorbance at 570 nm using a microplate reader. The representative ATP levels were normalized to total protein concentration.

### Identification of degenerating neurons using Fluoro-Jade B (FJB)

For detecting neuronal degeneration, brain tissue sections were stained with Fluoro-Jade B (FJB, Biosensis, Thebarton, SA, Australia). This staining was performed in accordance with the methods described by Choi et al. and others [39–41], after mounting the sections on gelatin-coated slides (Fisher Scientific, Pittsburgh, PA, USA). The staining protocol followed the referenced guidelines to ensure accurate identification of degenerating neurons. After staining, the tissues were mounted with DPX solution (Sigma-Aldrich Co., St. Louis, MO, USA) for preservation and clarity. The stained sections were then examined under a fluorescence microscope (BX53, Olympus, Shinjuku, Japan) using a FITC green filter (light wavelength: 450–490 nm) to identify FJB-positive cells. Counting of FJB-positive cells was conducted in a blinded manner, and the data were subsequently subjected to statistical analysis to evaluate the extent of neuronal degeneration.

### Immunofluorescence (IF) and immunohistochemistry (IHC) staining

For IF staining, brain tissues underwent washing with 0.01 M PBS and was pretreated with 1.2% hydrogen peroxide for 15 min to block endogenous peroxidase activity. Afterward, tissues were incubated with primary antibodies diluted in PBS containing 0.3% Triton X-100 for overnight incubation. This step was followed by staining with appropriate secondary antibodies for 2 hr. The stained brain sections were then mounted using DPX solution (Sigma-Aldrich Co., St. Louis, MO, USA) and examined using a BX53 fluorescence microscope (Olympus, Shinjuku, Japan). For IHC staining, after staining with primary antibody NeuN (diluted 1:500, Billerica, Millipore Co., Burlington, MA, USA), the tissues were second anti-rabbit IgG antibody (diluted 1:250, Jackson Immunoresearch lac, West Grove, PA, USA) for 2 hr at RT. The tissues were then stained with ABC solution (Vector Laboratories, Burlingame, Vector, CA, USA) for 2 hr at RT, followed by DAB (0.06% 3, 3′-diaminobenzidine solution, Sigma-Aldrich Co., St Louis, MO, USA) for 1 min 30 s. Intensity measurements were conducted using the Image J software (NIH, Bethesda, Rockville, MD, USA). Detailed information about the antibodies used can be found in the supplementary.

### Behavioral test

#### Modified neurological severity score (mNSS) test

To evaluate neurological motor function, mNSS tests were performed after inducing TBI and administering lactate. We performed this mNSS test 8 times, once a day for 4 hr, 1, 2, 3, 4, 5, 6 and 7 days. Each mice in the experiment were evaluated for its inability to walk, balance, startle, disorientation, and inability to exit a circle. Mice receive a score of 0 if they do the test or 1 if they don’t, so a total score closer to 10 indicates severe motor dysfunction [42].

#### Morris water maze (MWM) test

To evaluate cognitive function, MWM tests were performed after mNSS test. We performed the MWM test 4 times, once a days for 8, 9, 10, and 11 days after TBI. Each mice swam 4 times a day in a circular tank of water, starting in each quadrant. The mice swam for a total of 120 s until they found the hidden platform. The hidden platform was set at 1 cm below the water surface in the center of the first quadrant. We recorded using a camera and evaluated with SMART video tracking software 3.0 (Panlab, Carrer del’Energia, Spain) [43].

### Statistical analysis

The experimental data were analyzed using a blind testing approach to minimize researcher bias. Quantitative results are presented as the mean ± standard error of the mean (SEM). Statistical significance was set at a p-value of less than 0.05. Non-parametric tests between groups were compared using the Mann–Whitney U test or Kruskal-Wallis test with post-hoc Bonferroni correction, and behavioral tests were assessed by analysis of variance (ANOVA). Statistical analyses were used the IBM Statistical Package for the Social Sciences (IBM SPSS Statistics version 26, Chicago, IL, USA) software.

## Supplementary information

Antibody information

Original western blot membrane

## Data Availability

All experimental datasets throughout the current study are available on rationale request to corresponding author.
