# Supplementary material for: Effects of astrocytic PKM2 gene deletion on neuronal death following traumatic brain injury
Source: Cell Death Discov. 2025 Nov 10;11:525. doi: 10.1038/s41420-025-02829-7 (PMC12603202; doi:10.1038/s41420-025-02829-7)
Supplement: Supplementary file 1 — Antibody information [file 41420_2025_2829_MOESM1_ESM.pdf]

Antibody	Target Species	Isotype	Dilution	Company (Cat No.)
PKM2	Rabbit	IgG	IF-1:500	Cell signaling (D78A4)
GFAP	Goat	IgG	IF-1:1000	Abcam (ab53554)
4HNE	Mouse	IgG	IF-1:500	Alpha Diagnostic Intl. lnc. (HNE13-M)
MAP2	Rabbit	IgG	IF-1:200	Abcam (ab32454)
LDHA	Rabbit	IgG	IF-1:100	Sigma-Aldrich Co. (SAB5700695)
MCT4	Mouse	IgG	WB-1:1000	Santa Cruz Biotechnology (sc-376140)
p-STAT3	Rabbit	IgG	WB-1:1000	Cell signaling (D3A7)
LDHB	Mouse	IgG	IF-1:100	Sigma-Aldrich Co. (WH0003945M1)
MCT2	Rabbit	IgG	IF-1:1000	Invitrogen (PA5-77498)
NeuN	Rabbit	IgG	IHC-1:500	Millipore (ABN78)
NeuN	Mouse	IgG	IF-1:500	Millipore (MAB377)
HIF-1α	Rabbit	IgG	WB-1:1000	GeneTex (GTX127309)
β-actin	Mouse	IgG	WB-1:10,000	Cell signaling (8H10D10)
Alexa Fluor 594	Rabbit	IgG	IF-1:250	Invitrogen (R37119)
Alexa Fluor 594	Mouse	IgG	IF-1:250	Invitrogen (A-11005)
Alexa Fluor 488	Goat	IgG	IF-1:250	Invitrogen (A-11055)
Alexa Fluor 488	Rabbit	IgG	IF-1:250	Invitrogen (A-21206)
Alexa Fluor 488	Mouse	IgG	IF-1:250	Invitrogen (A-21202)
Alexa Fluor 647	Mouse	IgG	IF-1:250	Invitrogen (A-21235)
