# Supplementary figures and images for: Effects of astrocytic PKM2 gene deletion on neuronal death following traumatic brain injury
Source: Cell Death Discov. 2025 Nov 10;11:525. doi: 10.1038/s41420-025-02829-7 (PMC12603202; doi:10.1038/s41420-025-02829-7)

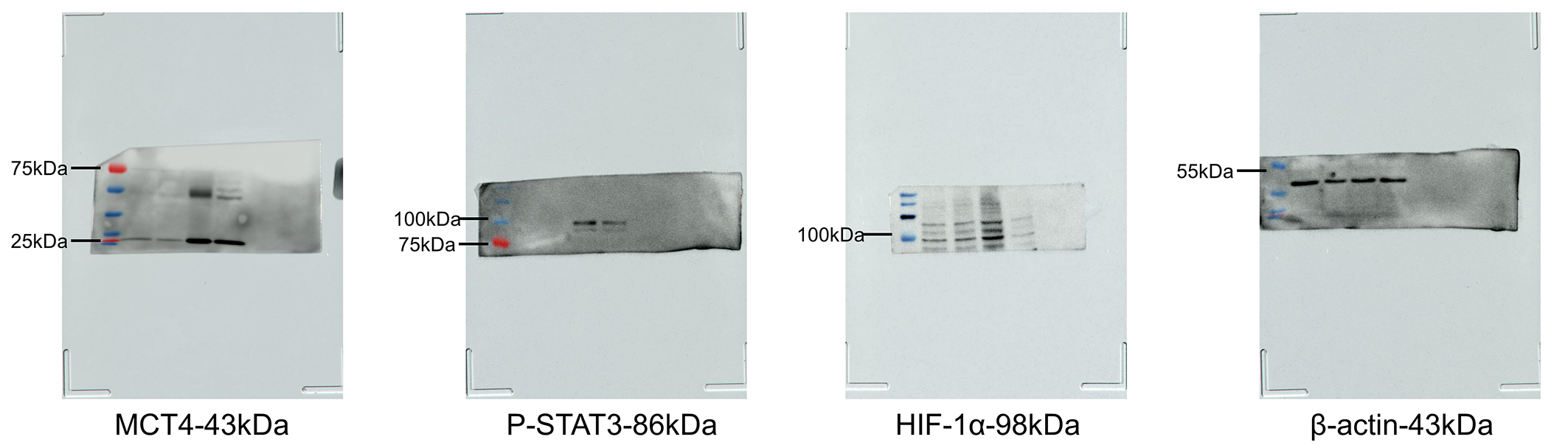

Supplement: Supplementary file 2 — Original western blot membrane [file 41420_2025_2829_MOESM2_ESM.tif]
